# Determining barriers to creating an enabling environment in Cambodia: results from a baseline study with key populations and police

**DOI:** 10.7448/IAS.19.4.20878

**Published:** 2016-07-18

**Authors:** Mira L Schneiders, Amy Weissman

**Affiliations:** 1 Mercator Fellow on International Affairs 2012/2013 with FHI 360, Phnom Penh, Cambodia; 2 Save the Children, Vientiane, Lao PDR (Formerly with FHI 360, Phnom Penh, Cambodia)

**Keywords:** key populations, enabling environment, police, law enforcement, harm reduction, drugs, sex work, HIV

## Abstract

**Introduction:**

Cambodian law enforcement's limited acceptance of harm reduction has hindered HIV program effectiveness. With funding from the Australian Department of Foreign Affairs and Trade, FHI 360 supported the Ministry of Interior to implement the Police Community Partnership Initiative (PCPI) in Cambodia's capital Phnom Penh. To guide this, FHI 360 conducted a baseline study examining police and key populations’ attitudes and practices towards one another, including fear and occurrence of arrest.

**Methods:**

Between December 2012 and January 2013, a cross-sectional survey of 199 police post officers, 199 people who use drugs (PWUD) including people who inject drugs (PWID), 199 men who have sex with men (MSM), 200 transgender women (TGW) and 200 female entertainment workers (FEW) was conducted in five Phnom Penh districts. Eligible participants were ≥18 years, members of a key population from selected hotspots or police officers, deputy chiefs or chiefs.

**Results:**

Key populations’ median age was 25 years (IQR: 22–30); 40% had completed only primary school. Police were male (99.5%), with median age 43 years (IQR: 30 to 47), and 45 and 25% high school and university completion rates, respectively. Key populations feared arrest for carrying needles and syringes (67%), condoms (23%) and 19% felt afraid to access health services. Close to 75% of police reported body searching and 58% arresting key populations in the past six months for using drugs (64%), selling or distributing drugs (36%) or being violent (13%). Self-reported arrests (23% PWUD, 6% MSM, 6% TGW, 12% FEW; *p*<0.05), being verbally threatened (45% PWUD, 21% MSM, 25% TGW, 27% FEW; *p*<0.001) and body searched (44% PWUD, 28% MSM, 23% TGW, 8% FEW; *p*<0.001) was significantly higher among PWUD than other key populations. The majority (94%) of police believed arrest was an appropriate solution to reduce HIV and drug use and reported selling sex (88%) and carrying needles and syringes (55%) as valid reasons for arrest.

**Conclusions:**

Key populations’ fear of accessing harm reduction and health services and police's negative attitudes and practices towards key populations present major barriers to HIV prevention efforts in Cambodia. To create an enabling environment and ensure police are allies in the Cambodian HIV response, interventions should tackle underlying negative attitudes among police towards key populations and vice versa.

## Introduction

Certain laws and policies and the manner in which they are implemented and enforced are known to hinder the delivery of HIV services and programmes and increase key populations’ susceptibility to HIV and other health related harms [1]. Negative impacts of punitive drug policies and related human rights abuses are particularly experienced by people who use drugs (PWUD) and sex worker populations [2–4]. A study among people who inject drugs (PWID) in Thailand found police beatings independently associated with drug-related harms [5].

In Cambodia, current laws and policies hamper the HIV response, reflected in the highest HIV prevalence being found among key populations – female entertainment workers (FEW): 23.1% [6], men who have sex with men (MSM): 2.2% [7], transgender women (TGW): 4.15% [8] and PWID: 24.4% [9]. Following the introduction of the 2008 Law on the Suppression of Human Trafficking and Sexual Exploitation, female entertainment and sex workers reported reduced mobility and reduced access to outreach and facility services and condoms [10]. By 2009, the establishment of 14 compulsory drug treatment/detention centres operated by the Military Police of the Royal Cambodian Armed Forces, the Ministry of Interior or the Ministry of Social Affairs, Veterans and Youth Rehabilitation led to the forcible detention of PWUD, without the provision of health care or drug-related services [11]. According to one study, 30% of methamphetamine users were forcibly sent to a centre by local authorities, with the median stay 90 days [11]. The 2010 Village/Commune Safety Policy, focused on “cleaning” Cambodia's streets, resulted in increased harassment, detention or arrest [12]. Following its introduction in 2011, drug-related arrests quadrupled and prison populations grew approximately 14% annually, with national prisons reaching 179% over capacity [13]. Prisons are among the highest-risk environments for HIV transmission [14], and the high rates of drug-related imprisonment seen in Cambodia further exacerbate HIV risks.

The World Health Organization (WHO) *Guidelines for the psychosocially assisted pharmacological treatment of opioid dependence* recommend wide accessibility of opioid dependence treatment as a minimum requirement for health systems at national level, including delivery in primary care settings [15]. In July 2010, Cambodia's only methadone maintenance treatment (MMT) programme was established in Phnom Penh by governmental and non-governmental (NGO) partners, with 61 heroin users enrolled by October 2010 [16]. From 2010 to 2014, according to service records, 432 patients were enrolled in the programme, with an average of 130 clients receiving a daily dose [17]. According to unpublished reviews of the programme, the lack of take-home doses or satellite clinics may have contributed to what routine data show as frequent drop-out and re-enrolment of clients.

Widespread consensus exists among government, UN agencies, NGOs and health and social service providers that in order to halt national HIV epidemics an enabling environment must be created to support HIV prevention and treatment while also ensuring law enforcement and public safety [12,18]. Therefore, to prevent further spread of HIV in Cambodia, as is the aim of the National Centre for HIV/AIDS, Dermatology and STD Control [7], it is critical to create an enabling environment in which key populations are able to access HIV and drug-related prevention, care and treatment.

Towards this effort, AusAID (now called the Department of Foreign Affairs and Trade)/the HIV/AIDS Asia Regional Program (HAARP) funded FHI 360 to implement the Police Community Partnership Initiative (PCPI), in the capital city Phnom Penh, which is home to the majority of the country's key populations. PCPI was previously implemented in Banteay Meanchay, Cambodia and aims to strengthen collaborative partnerships among Ministry of Interior (MoI) Provincial AIDS Committee and Secretariat, local authorities, police, health care providers, development partners, NGOs and members of key populations. Facilitating a harm reduction approach, in which police link key populations to needed services rather than detain or arrest them, PCPI entails regular coordination meetings and sensitization workshops at community level, capacity building, facilitated dialogue and problem solving among participating stakeholders.

Given that PCPI had not been previously evaluated, and given the dearth of monitoring or evaluation of harm reduction in Cambodia [19], a baseline study was conducted prior to implementing PCPI in Phnom Penh [20]. The objective of this baseline was to gather data to serve as a measure for assessing the effectiveness of the PCPI approach. The study measured police officers’ attitudes and practices towards key populations and their service providers (including frequency and occurrence of arrests, detainment and referrals) and key populations’ HIV-related practices and attitudes towards police (including fear of and occurrence of arrests). Because of the early closure of HAARP, an end line study was not conducted. Nonetheless, this study provides important insights into law enforcement practices in Cambodia by identifying critical barriers that must be overcome for an effective harm reduction approach to be implemented and can thus inform future work in this area.

## Methods

Following approval by the National Ethics Committee for Health Research (NECHR) of Cambodia and FHI 360's Protection of Human Subjects Committee in 2012, data were collected in five Khans (districts) of Phnom Penh where PCPI would be implemented. Structured interviews were conducted one-on-one using paper-based questionnaires with questions related to attitudes as well as experience of arrest and harassment adapted to each study population: police post officers, PWUD (including PWID), MSM, TGW and FEW. To assure data accuracy, questionnaire data were double entered into Epidata, and statistical analysis was conducted using STATA Version 12.0.

### Sampling procedures

Participants from the four key population groups were selected using multistage sampling. Hotspots, places where key populations are known to work or gather (such as parks, riverbanks, bars and night clubs, massage parlours, beer gardens, karaoke clubs, rented rooms or abandoned buildings) in the five districts were chosen using cluster random sampling for each group by random number allocation via Randomize software. A list of hotspots for each group was created during a consultation meeting with organizations working in the districts. Because the number of identified hotspots for PWUD was limited, the sample was drawn from all hotspots and thus was not random. At each hotspot, convenience sampling was used to select participants.

Police were also selected using a multistage sampling procedure. Cluster random sampling was used to select 20 out of 46 existing police posts in the five study districts. At each post, convenience sampling was then used to select a maximum of 10 police officers, one who had to be a chief and one a vice-chief of police.

Sample size calculations were based on the assumption that the key indicator for key populations’ attitudes towards police (fear of arrest), would decrease from an estimated 50% at baseline, to 30% after one year of PCPI implementation. Using Epi-Info's STATCAL, the sample size, including a 10% assumed refusal rate, was calculated as 200.

### Eligibility

All participants had to be 18 years or older, Khmer-speakers and able and willing to provide oral informed consent. The election date during the UN Transitional Authority in Cambodia (UNTAC) was used to verify participants were 18 years or older. Participants reporting having being born after the UNTAC election date would be less than 18 years old and were excluded. Police officers had to be working at the respective post at the time of the study. Additionally, PWUD had to report use of any illicit drug as defined by Cambodian law by any route (injection or non-injection) at least two times in the past six months. FEW had to be biologically female and have been an entertainment worker working in any venue or non-venue type in the past six months. MSM had to be biologically male and reported having sex with a biological male in the past six months. TGW had to be biologically male at birth and self-identify and/or express themselves as a different gender from their biological sex at birth.

### Study procedures

All participants provided oral informed consent before the interview. One-on-one interviews were conducted at the recruitment sites, including police posts, if confidentiality and safety could be ensured. Otherwise, transportation to a nearby NGO-run drop-in-centre was provided. Participants were assigned a personal identification number used on questionnaires with no personal identifying information linked to responses. Trained interviewers worked in teams of two, with female interviewers interviewing FEW, and male interviewers interviewing remaining participants. Each interview lasted approximately 15 minutes and participants received a gift worth 1.5 USD for their time.

For key populations, prior to conducting surveys, venue owners were asked permission to interview their workers. This did not apply to public places, such as parks and streets. At each hotspot, interviewers approached individuals presenting at the site to explain the study. Those willing to participate were then screened for eligibility, consented and interviewed.

For police, a formal letter from the MoI informed the Commissariat Police of Phnom Penh about the study. The commissioner subsequently informed the chiefs of selected police post offices and the research study coordinator, presenting the MoI letter and NECHR approval. On the interview day, police chiefs informed officers and read them a statement about the study, highlighting that participation was voluntary and would not affect job responsibility, promotion or benefits. Police officers consenting to participate were interviewed by one researcher.

## Results

### Socio-demographic characteristics

#### Key populations

In total, 798 individuals (200 FEW, 199 MSM, 200 TGW and 199 PWUD) participated in this study, the majority of whom were male (*n*=542, 67.9%), younger (the median age 25 years, IQR: 22 to 30), educated and employed (Table 1). Primary school was completed by 39.4% of key population participants. MSM were the most educated group with 36.2% (data not shown) and 18.1% completing grade 12 or university and above, respectively. PWUD had the highest rates of never attending school (17.1%).

**Table 1:** Socio-demographic characteristics of key populations

	FEW (*n*=200)	MSM (*n*=199)	PWUD (*n*=199)	TGW (*n*=200)	Total (*n*=798)
Gender: male (%)	0 (0.0)	199 (100.0)	143 (71.9)	200 (100.0)^a^	542 (67.9)^a^
Mean age*	26.9	25.8	28.0	26.1	26.7
Median age (IQR)	25 (21 to 31)	24 (21 to 27)	27 (23 to 31)	24 (21 to 29)	25 (22 to 30)
Level of education completed (%)**					
Never attended school	25 (12.5)	1 (0.5)	34 (17.1)	2 (1.0)	62 (7.8)
Primary school (year 1–6)	133 (66.5)	30 (15.1)	121 (60.8)	30 (15.0)	314 (39.4)
Lower and upper secondary school (year 7–12)	67 (33.5)	133 (66.8)	76 (38.2)	138 (69.0)	414 (51.9)
University and above	0 (0.0)	36 (18.1)	2 (1.0)	32 (16.0)	70 (8.8)
Main source of income (%)					
Student	0 (0.0)	33 (16.6)	0 (0.0)	14 (7.0)	47 (5.9)
Service job^b^	3 (1.5)	75 (37.7)	27 (13.6)	117 (58.5)	222 (27.8)
Manual labour^c^	0 (0.0)	15 (7.5)	37 (18.6)	13 (6.5)	65 (8.2)
Office job/business owner	1 (0.5)	22 (11.1)	14 (7.0)	20 (10.0)	57 (7.1)
Seller	0 (0.0)	21 (10.6)	12 (6.0)	20 (10.0)	53 (6.6)
Garbage collector/beggar	0 (0.0)	1 (0.5)	62 (31.2)	0 (0.0)	63 (7.9)
Entertainment venue based EW^d^	151 (75.5)	1 (0.5)	10 (5.0)	1 (0.5)	163 (20.4)
Street based, brothel based, home based and freelance EW	45 (22.5)	0 (0.0)	6 (3.0)	0 (0.0)	51 (6.4)
Male/MSM/TGW sex worker	0 (0.0)	14 (7.0)	0 (0.0)	4 (2.0)	18 (2.3)
Unemployed	0 (0.0)	16 (8.0)	31 (15.6)	11 (5.5)	58 (7.3)
Other	0 (0.0)	1 (0.5)	0 (0.0)	0 (0.0)	1 (0.1)
Used any drug in past three months (%)	21 (10.5)	37 (18.6)	198 (100.0)	5 (2.5)	261 (32.8)
Crystal, ice (methamphetamine)**	20 (95.2)	36 (97.3)	192 (97.0)	4 (80.0)	252 (96.6)
Yama (amphetamine)**	6 (28.6)	8 (21.6)	52 (26.3)	0 (0.0)	66 (25.3)
Heroin/opium**	1 (4.8)	2 (5.4)	44 (22.2)	0 (0.0)	47 (18.0)
Inhalants (e.g. glue, paint, and petrol)**	0 (0.0)	2 (5.4)	18 (9.1)	0 (0.0)	20 (7.7)
Marijuana**	1 (4.8)	0 (0.0)	10 (5.1)	0 (0.0)	11 (4.2)
Ketamine	1 (4.8)	1 (2.7)	5 (2.5)	2 (40.0)	9 (3.4)
Had sex, among those who used any drug in past three months (%)	21 (100.0)	37 (100.0)	162 (81.4)	5 (100.0)	225 (86.2)
Used drugs before or during sex	15 (71.4)	27 (73.0)	108 (66.7)	3 (60.0)	153 (68.0)
Exchanged sex for drugs**	1 (4.8)	13 (35.1)	13 (8.0)	1 (20.0)	28 (12.4)

* Statistically significant to *p*<0.05

** statistically significant to *p*<0.001

a refers to gender at birth

b such as hair dresser, waiter, cleaner, driver and security guard

c such as construction worker, factory worker and mechanic

d such as beer garden EW, beer promoter, massage parlour/spa FEW and café FEW.

The majority of key population participants were employed in service jobs (27.8%) (Table 1). Three-fourths of FEW earned income by working at entertainment venues, with close to one-fourth being street based, brothel based, home based or freelance. Among TGW and MSM, service jobs were the most common source of income (58.5 and 37.78%, respectively). Low wage jobs predominated among PWUD (manual labourer: 18.6%; garbage collector/beggar: 31.2%) with unemployment among PWUD higher (15.6%) than among other groups.

Rates of drug use within the past three months varied significantly among non-PWUD participants, with MSM having significantly higher rates of drug use (18.6%) than FEW (10.5%) and TGW (2.5%), *p*<0.05 (Table 1). Among PWUD, 17.1% (*n*=34) injected drugs. Drug use before or during sex was most common among MSM (73.0%) and FEW (71.4%).

#### Police

In total, 199 police participated in the study, the majority of who were male (99.5%). Police participants had a median age of 43 years (IQR: 30 to 47) and were educated (Table 2). Approximately 45% completed at least high school (data not shown), with one-fourth having attended university and above. The majority of police participants worked as police post officers (77.4%), whereas 19.1% were deputy chiefs and 3.5% were chief of police. On average, police participants worked at their respective post for 10.2 years.

**Table 2:** Socio-demographic characteristics of police

	Police post officers (*n=*199)
Gender: male (%)	198 (99.5)
Mean age	39.6
Median age (IQR)	43 (30 to 47)
Level of education completed	
Primary school (year 1 to 6) (%)	2 (1.0)
Lower and upper secondary school (year 7 to 12) (%)	147 (73.9)
University and above (%)	50 (25.1)
Current position	
Police post officer (%)	154 (77.4)
Deputy chief of police post office (%)	38 (19.1)
Chief of police post office (%)	7 (3.5)
Mean duration working in current office (years)	10.2
Median years (IQR)	5 (1.3 to 20)

## Harassment and arrest

### Key populations

More than 11% of key population participants reported being arrested in the past six months, with arrest rates and reasons for arrest differing among groups. PWUD reported arrest at a significantly higher rate than FEW and MSM (Fisher's exact test: *p*<0.05) (Table 3). The most common reason for last arrest among all groups was using drugs (32.9%), selling sex (27.1%) and being violent (16.4%) (Table 4). Significantly more PWUD than MSM and TGW were arrested for using drugs (54.3% vs. 7.7% and 54.3% vs. 6.7%, respectively) (*p*<0.001), whereas 100% of arrested FEW were arrested for selling sex, significantly higher than among all other groups (*p*<0.001). Arrests among MSM (46.2%) and TGW (46.7%) primarily related to engaging in violent behaviour.

**Table 3:** Experience of arrest among key populations in past six months

	FEW (*n=*200)	MSM (*n*=199)	PWUD (*n*=199)	TGW (*n=*200)	Total (*n*=798)
Arrests (%)*	24 (12.0)	12 (6.0)	46 (23.1)	12 (6.0)	94 (11.8)
Mean number of arrests	1.2	1.3	1.8	1.2	1.5
Median number of arrests (IQR)	1 (1 to 1)	1 (1 to 1.5)	1 (1 to 2)	1 (1 to 1)	1 (1 to 2)

* Statistically significant to *p*<0.05.

**Table 4:** Reasons for last arrest and experience of being arrested at last arrest

	FEW (*n*=31)	MSM (*n*=13)	PWUD (*n=*81)	TGW (*n*=15)	Total (*n*=140)
Reason for last arrest (%)					
Drug use**	0 (0.0)	1 (7.7)	44 (54.3)	1 (6.7)	46 (32.9)
Selling sex**	31 (100.0)	1 (7.7)	2 (2.5)	4 (26.7)	38 (27.1)
Conducting violence**	0 (0.0)	6 (46.2)	10 (12.4)	7 (46.7)	23 (16.4)
Theft/robbery*	0 (0.0)	0 (0.0)	16 (19.8)	1 (6.7)	17 (12.1)
Sleep in public space	0 (0.0)	0 (0.0)	4 (4.9)	0 (0.0)	4 (2.9)
Selling, distributing, buying or carrying drugs	0 (0.0)	2 (15.4)	5 (6.2)	0 (0.0)	7 (5.0)
Other**	0 (0.0)	4 (30.8)	3 (3.7)	7 (46.7)	14 (10.0)
Experience at last arrest (%)					
Detained at police station; then released by police within 48 hours	21 (67.7)	10 (76.9)	40 (49.4)	9 (60.0)	80 (57.1)
Referred to centre for social affairs (without sending to court)	4 (12.9)	2 (15.4)	10 (12.4)	1 (6.7)	17 (12.2)
Detained at police station; then referred to rehab centre (without sending to court)*	1 (3.2)	0 (0.0)	14 (17.3)	0 (0.0)	15 (10.7)
Arrested and released immediately at site in exchange for money	1(3.2)	0 (0.0)	9 (11.1)	2 (13.3)	12 (8.6)
Arrested and released immediately at site in exchange for sex	0 (0.0)	0 (0.0)	0 (0.0)	1 (6.7)	1 (0.7)
Detained at police station; then convicted by court and sent to prison	0 (0.0)	0 (0.0)	6 (7.4)	1 (6.7)	7 (5.0)
Referred directly to MMT clinic (without sending to court)	1 (3.2)	1 (7.7)	0 (0.0)	0 (0.0)	2 (1.4)
Dropped outside of Phnom Penh	0 (0.0)	0 (0.0)	2 (2.5)	0 (0.0)	2 (1.4)
Other*	3 (9.7)	0 (0.0)	0 (0.0)	1 (6.7)	4 (2.9)

* Statistically significant to *p*<0.05

** statistically significant to *p*<0.001.

Among key population participants who reported arrest in the past year, more than half (57.1%) were detained at the police station and released within 48 hours (Table 4). A smaller proportion were referred to a social affairs (12.2%) or rehabilitation centre (10.7%) (the former is a voluntary centre for all populations; the latter is designed for PWUD and may be voluntary or involuntary with no formal criteria to determine length of stay and only abstinence-based treatment [21]) without being sent to court, which was significantly more common among PWUD than FEW (17.3% vs. 3.2%, respectively, *p*<0.05). Overall, the majority of arrests did not result in being sent to court. Only 5.0% of arrested key population participants reported being sent to prison following a court conviction.

Financial incentives were sometimes used to negotiate with police. Close to 10% of key population participants reported having exchanged money in return for immediate release at last arrest (Table 4), whereas 7.6% were forced to pay money to avoid arrest or harassment (Table 5). Exchange of sex to negotiate immediate release following arrest (Table 4) or to avoid arrest or harassment (Table 5) was rare to none (0.7 and 2.3%, respectively). More FEW were forced to pay money or exchange sex to avoid arrest than other groups (12.5 and 5.0%, respectively, *p*<0.05).

**Table 5:** Experience of threat, body search or coercion by police in past six months

	FEW (*n*=200)	MSM (*n=*199)	PWUD (*n*=199)	TGW (*n*=200)	Total (*n*=798)
Verbally threatened (%)**	53 (26.5)	41 (20.6)	89 (44.7)	50 (25.0)	233 (29.2)
Body searched for condoms, needles/syringes and/or illicit drugs (%)**	16 (8.0)	56 (28.1)	88 (44.2)	46 (23.0)	206 (25.8)
Forced to pay money to avoid arrest or harassment (%)*	25 (12.5)	12 (6.0)	18 (9.0)	6 (3.0)	61 (7.6)
Forced to exchange sex to avoid arrest or harassment (%)*	10 (5.0)	5 (2.5)	1 (0.5)	2 (1.0)	18 (2.3)

* Statistically significant to *p*<0.05

** statistically significant to *p*<0.001.

Nearly one-third (29.2%) of key population participants reported being verbally threatened by police or local authorities in the past six months, with significantly more threats reported by PWUD than other groups (44.7%, *p*<0.001) (Table 5). Being body searched by police in the past six months was reported by one-fourth of all key population participants, significantly higher among PWUD (44.2%, *p*<0.05; among these 19.3% PWID, *n*=17), whereas the proportion of body searched FEW was the lowest (8.0%, *p*<0.001).

All key population participants reported experiencing fear of arrest in the past six months (Table 6). Overall, 7.0% of key population participants reported having relocated out of fear of interacting with police, whereas 4.6% hid from outreach workers fearing identification by police.

**Table 6:** Risk behaviours and related experience of fear of arrest in the past six months

	FEW (*n=*200)	MSM (*n*=199)	PWUD (*n*=199)	TGW (*n*=200)	Total (*n*=798)
Carried needles and syringes (%)	1 (0.5)	8 (4.0)	38 (19.1)	7 (3.5)	54 (6.8)
Fear of arrest for carrying N&S (%)	1 (100.0)	6 (75.0)	25 (65.8)	4 (57.1)	36 (66.7)
Carried condoms (%)	55 (27.5)	154 (79.9)	103 (51.8)	154 (77.0)	471 (59.0)
Fear of arrest for carrying condoms (%)*	12 (21.8)	11 (6.9)	16 (15.5)	16 (10.4)	55 (11.7)
Sold sex (%)	108 (54.0)	112 (56.3)	35 (17.6)	105 (52.5)	360 (45.1)
Fear of arrest for selling sex (%)*	47 (43.5)	30 (26.8)	15 (42.9)	28 (26.7)	120 (33.3)
Used drugs (%)	23 (11.5)	41 (20.6)	199 (100.0)	11 (5.5)	274 (31.0)
Fear of arrest for being a drug user (%)*	13 (56.5)	32 (78.1)	163 (81.9)	6 (54.6)	214 (78.1)
Being a MSM (%)	–	199 (100.0)	5 (2.5)	–	204 (25.6)
Fear of arrest for being a MSM (%)	–	60 (30.2)	1 (20.0)	–	61 (29.9)
Being a TGW (%)	–	–	0 (0.0)	200 (100.0)	200 (100.0)
Fear of arrest for being a TGW (%)	–	–	0.0	52 (26.0)	52 (26.0)
Relocated due to fear of interaction with police** (%)	16 (8.0)	8 (4.0)	30 (15.1)	2 (1.0)	56 (7.0)
Hidden from outreach workers due to fear of identification by police in past six months (%)	6 (3.0)	12 (6.0)	7 (3.5)	12 (6.0)	37 (4.6)

* Statistically significant to *p*<0.05; –Not applicable (this group was not surveyed for respective questions).

The majority of key population participants (60.5%), particularly PWUD (75.4%), felt police were unkind (Table 7). One-fourth of key population participants felt afraid of carrying condoms, with half of FEW expressing such fears. Approximately 20 and 27% of all key population participants were afraid of accessing health and legal services, respectively.

**Table 7:** Key populations’ attitudes towards police, harm reduction and other services

	FEW (*n=*200)	MSM (*n*=199)	PWUD (*n*=199)	TGW (*n*=200)	Total (*n*=798)
Police are kind to people like me (%)					
Strongly agree/agree	96 (48.0)	77 (38.7)	49 (24.6)	67 (33.5)	289 (36.2)
Strongly disagree/disagree	94 (47.0)	114 (57.3)	150 (75.4)	125 (62.5)	483 (60.5)
Do not know	10 (5.0)	8 (4.0)	0 (0.0)	8 (4.0)	26 (3.3)
I am afraid of carrying condoms (%)					
Strongly agree/agree	98 (49.0)	25 (12.6)	39 (19.6)	23 (11.5)	185 (23.2)
Strongly disagree/disagree	102 (51.0)	172 (86.4)	160 (80.4)	172 (86.0)	606 (75.9)
Do not know	0 (0.0)	2 (1.0)	0 (0.0)	5 (2.5)	7 (0.9)
I am afraid to access health services (HIV and STI testing, family planning/reproductive health) (%)					
Strongly agree/agree	34 (17.0)	54 (27.1)	22 (11.1)	44 (22.0)	154 (19.3)
Strongly disagree/disagree	166 (83.0)	143 (71.9)	177 (88.9)	154 (77.0)	640 (80.2)
Do not know	0 (0.0)	2 (1.0)	0 (0.0)	2 (1.0)	4 (0.5)
I fear accessing legal services (%)					
Strongly agree/agree	41 (20.5)	60 (30.2)	49 (24.6)	66 (33.0)	216 (27.1)
Strongly disagree/disagree	159 (79.5)	137 (68.8)	150 (75.4)	132 (66.0)	578 (72.4)
Do not know	0 (0.0)	2 (1.0)	0 (0.0)	2 (1.0)	4 (0.5)

#### Police

The majority of police (58.2%) reported arresting a member of a key population in the past six months (Table 8). Primary reasons related to using (63.8%) or selling or distributing (36.2%) drugs. PWUD (83%) were most commonly arrested, followed by FEW (25.5%), TGW (12.8%) and >1% of arrests MSM (data not shown). Body searching a key population in the past six months for condoms, needles, syringes and/or illicit drugs was reported by close to three-fourths of police. Although following outreach workers to find and arrest key populations was rare (3%), 21.1% of police reported disrupting outreach in their patrol area in the past six months (data not shown).

**Table 8:** Arrest of an individual belonging to a key population group in past six months and reasons for last arrest

	Police (*n*=141)
Key populations arrests in past six months (%)	116 (58.2)
Mean number of arrests	4.6
Median number of arrests (IQR)	2 (1–4)
Reason for last arrest (%)	
Drug use	90 (63.8)
Selling sex	1 (0.7)
Conducting violence	19 (13.5)
Theft/robbery	12 (8.5)
Sleep in public space	7 (5.0)
Selling/distributing drugs	51 (36.2)
Carrying condoms	1 (0.7)
Other	6 (4.2)

Nearly half of police participants (46%, *n*=65) reported referring the arrestee to the Khan police after their last arrest. Fifteen percent (*n*=15) reported that they sent the individual to court or directly referred him or her to a rehabilitation centre after detainment at the police station. Thirteen percent (*n*=18) reported releasing the arrestee within 48 hours after detainment at the police station, and 10% (*n*=14) directly referred the individual to the centre for social affairs. No police participants reported making a referral to the MMT clinic [16]. Immediately releasing the arrested individual at the site of arrest was reported by only 1% (*n*=2) of police.

All police (100%) reported supporting the provision of HIV prevention and harm reduction, yet, the majority (94.0%) thought arrest and detention an appropriate solution for reducing HIV transmission and drug use (Table 9). Furthermore, the majority of police reported that drug use in private spaces (96.5%), selling sex (88.4%) and carrying needles and syringes (55.2%) were valid reasons for arrest.

**Table 9:** Police attitudes towards key populations and harm reduction

	Police (*n=*199)
Arresting and detaining key populations are appropriate solutions for reducing HIV/AIDS and drug use (%)	
Strongly agree/agree	187 (94.0)
Strongly disagree/disagree	11 (5.5)
Do not know	1 (0.5)
There is good collaboration between the community and police for HIV and harm reduction programming (%)	
Strongly agree/agree	198 (99.5)
Strongly disagree/disagree	1 (0.5)
I support the provision of HIV prevention and harm reduction in my community	
Strongly agree/Agree	199 (100.0)
Police should arrest key populations because of the following reasons (%)	
Using drugs in private places	192 (96.5)
Selling sex	176 (88.4)
Carrying N&S	110 (55.2)
Carrying condoms	4 (2.0)

## Discussion

This study established a baseline of harm reduction related attitudes and practices among key population groups and police in five Phnom Penh districts. Similar to experience in other settings [2,3], our results indicate that current punitive laws and policies and their implementation have created an environment in which key populations in Cambodia are burdened by fear of and actual harassment and arrest.

Reasons for fearing police differed among key populations, suggesting that efforts to change attitudes need to be tailored to each group. PWUD not only feared arrest for carrying needles or syringes (66%), but also most commonly reported relocating because of fear of police, making this group hard to reach and impeding HIV outreach activities. Research in other settings, including Myanmar [22], demonstrate that drug control efforts targeting drug paraphernalia lead to transience of PWUD populations and increased needle sharing, thus accelerating HIV transmission [23]. These findings have important implications for Cambodia's approach to drug control.

Our study also found fear of arrest for carrying condoms, which was reported most often by FEW. Yet, when governments support condom use, for instance, as in the Dominican Republic, where the government sanctioned condom availability in establishments, and monitored establishments to ensure supplies, there is a positive influence on consistent condom use among FEW [24]. The fear identified in Cambodia likely reflects the negative influence of the 2008 legislation prohibiting sex work on prevention efforts, access to outreach, peer support and condom use among this population [10]. Furthermore, our findings indicate that the 2008 legislation impeded prevention efforts by undermining the 100% condom use programming, which was attributed with supporting a decline in HIV transmission in Cambodia (and other neighbouring countries) [25].

A recent review identified structural barriers, systematic discrimination and violence because of gender identity, as a contributing factor to HIV risk for TGW [26]. In our study, close to one-third and one-fourth of MSM and TGW, respectively, reported fear of arrest for their sexual or gender identity. This fear likely drives MSM and TGW individuals underground, inhibiting safer sex and drug use. It also reflects widespread stigma, discrimination and social exclusion, including from health services, as reported by lesbian, gay, bisexual and transgender persons in Cambodia, with TGW being most affected [27]. However, it is not only MSM and TGW who face consequences because of stigma and discrimination. Such consequences are common among all key populations who face stigma related to their identity, practices and/or occupation, and the criminalization or lack of legal protections for these identities, practices or occupations increases the risk of HIV acquisition [28].

Nearly all police (97%) believed arresting and detaining key populations was a solution for reducing HIV transmission and drug use and that arrests for selling sex (88%) and carrying needles/syringes (55%) were appropriate, confirming fears expressed by FEW and PWUD. Yet, all police voiced support of HIV prevention and harm reduction services in their community. This contradiction between attitudes and practice suggests a need to clarify how to operationalize and harmonize existing laws and policies and to foster understanding of the mutual benefits of harm reduction to HIV and law enforcement efforts. Interventions in the USA showed that even short training sessions can help police more effectively align occupational safety with public health goals [29].

Although 17% of key population participants reported having been arrested and 70% of police reported having arrested a key population member in the past year, there was a notable mismatch between the key populations’ reported rates of arrests for selling sex (27%) and corresponding rates reported by police (1%). Furthermore, this study provides no evidence of arrests being made for carrying condoms or needles or syringes. Nonetheless, levels of fear were high, making evident that internalized fear and negative attitudes towards police persist and must be mitigated. It is possible that such fear prevails because of key populations’ experience of harassment and discrimination, primarily among PWUD and FEW and body searching experienced by PWUD. This, combined with FEW being most commonly forced to pay money or exchange sex to avoid arrest or harassment, suggests that certain key populations are more vulnerable, likely due to laws and policies targeting specific behaviours they are engaged in.

Few key populations reported having been sent to prison following arrest. It is worth noting that this number is likely to underrepresent the true number of arrests, as sampling did not capture individuals detained in prison at the time of the study. However, among those ever imprisoned, only 5% were sent to court to be convicted. Furthermore, referrals to social services or MMT by police were rare to none and findings showed that police had poor understanding of the essential components of harm reduction. These findings suggest a need to improve awareness and knowledge of MMT and social referral mechanisms among police who are frequently in contact with high-risk individuals, and hence play an important role in bridging between key populations and harm reduction services.

Despite the lack of reliable evaluations of the MMT program, anecdotal evidence and service records indicate low overall uptake and high drop-out, re-enrolment and co-use of methadone treatment and drugs by injection among its recipients [30]. In line with WHO recommendations [15], these findings suggest an urgent need to improve accessibility of methadone treatment in Cambodia, such as by diversifying availability beyond the single MMT clinic (e.g. using satellite clinics). Assessments of Cambodia's rehabilitation centres show that these lack formal criteria determining length of stay, solely offer abstinence-based treatment and that HIV prevention is limited to information brochures [21]. Given the lack of alternative evidence-based treatment options for drug dependence in Cambodia, improving referral mechanisms and the quality of the single available MMT clinic in Cambodia is paramount.

Although this study contributes greatly to understanding the implications of law enforcement and policies on key populations, a number of limitations must be recognized. Study participants were only drawn from the capital, sampled MSM and TGW represent those who openly self-identify and not necessarily individuals who hide their sexual or gender identity, and PWUD represent only those from the coverage area of one organization. As such, sampled key populations may vary in meaningful ways from others in other parts of the country. Additionally, participants willing and able to participate may have differed from those who did not, for instance, by being more comfortable with and/or having more frequent contact with organizations and outreach workers. Finally, because of the relatively small number of subpopulations within the FEW (such as street based FEW) and PWUD samples (injecting and non-injecting drug users) this study could not determine possible differences within these groups.

## Conclusions

This study illustrates that key populations’ fear of accessing harm reduction and health services and police's negative attitudes and practices are key barriers to HIV prevention and treatment efforts in Cambodia. It further suggests that future efforts aiming to align law enforcement with HIV services and wider public health goals, such as PCPI, must address the underlying negative attitudes held by key populations and police. Such efforts should be tailored to each key population group and evaluated for effectiveness.

Findings from this study also bolster global recognition of the importance of an enabling environment for effective HIV programs and services. A paradigm shift from punitive laws and policies towards harm reduction and rights-based principles, as well as effective multi-sectoral collaboration are all imperative for reducing HIV in Cambodia and other countries with key population driven HIV epidemics.
